# Treatment with the mitochondrial‐targeted antioxidant peptide SS‐31 rescues neurovascular coupling responses and cerebrovascular endothelial function and improves cognition in aged mice

**DOI:** 10.1111/acel.12731

**Published:** 2018-02-06

**Authors:** Stefano Tarantini, Noa M. Valcarcel‐Ares, Andriy Yabluchanskiy, Gabor A. Fulop, Peter Hertelendy, Tripti Gautam, Eszter Farkas, Aleksandra Perz, Peter S. Rabinovitch, William E. Sonntag, Anna Csiszar, Zoltan Ungvari

**Affiliations:** ^1^ Reynolds Oklahoma Center on Aging University of Oklahoma Health Sciences Center Oklahoma City OK USA; ^2^ Translational Geroscience Laboratory Donald W. Reynolds Department of Geriatric Medicine University of Oklahoma Health Sciences Center Oklahoma City OK USA; ^3^ Division of Clinical Physiology Faculty of Medicine University of Debrecen Debrecen Hungary; ^4^ Department of Medical Physics and Informatics Faculty of Medicine and Faculty of Science and Informatics University of Szeged Szeged Hungary; ^5^ Arthritis & Clinical Immunology Research Program Oklahoma Medical Research Foundation Oklahoma City OK USA; ^6^ Department of Pathology University of Washington Seattle WA USA

**Keywords:** aging, cerebral circulation, endothelial dysfunction, oxidative stress, vascular cognitive impairment

## Abstract

Moment‐to‐moment adjustment of cerebral blood flow (CBF) via neurovascular coupling has an essential role in maintenance of healthy cognitive function. In advanced age, increased oxidative stress and cerebromicrovascular endothelial dysfunction impair neurovascular coupling, likely contributing to age‐related decline of higher cortical functions. There is increasing evidence showing that mitochondrial oxidative stress plays a critical role in a range of age‐related cellular impairments, but its role in neurovascular uncoupling remains unexplored. This study was designed to test the hypothesis that attenuation of mitochondrial oxidative stress may exert beneficial effects on neurovascular coupling responses in aging. To test this hypothesis, 24‐month‐old C57BL/6 mice were treated with a cell‐permeable, mitochondria‐targeted antioxidant peptide (SS‐31; 10 mg kg^−1 ^day^−1^, i.p.) or vehicle for 2 weeks. Neurovascular coupling was assessed by measuring CBF responses (laser speckle contrast imaging) evoked by contralateral whisker stimulation. We found that neurovascular coupling responses were significantly impaired in aged mice. Treatment with SS–31 significantly improved neurovascular coupling responses by increasing NO‐mediated cerebromicrovascular dilation, which was associated with significantly improved spatial working memory, motor skill learning, and gait coordination. These findings are paralleled by the protective effects of SS–31 on mitochondrial production of reactive oxygen species and mitochondrial respiration in cultured cerebromicrovascular endothelial cells derived from aged animals. Thus, mitochondrial oxidative stress contributes to age‐related cerebromicrovascular dysfunction, exacerbating cognitive decline. We propose that mitochondria‐targeted antioxidants may be considered for pharmacological microvascular protection for the prevention/treatment of age‐related vascular cognitive impairment (VCI).

## 1 INTRODUCTION

Normal functioning of the central nervous system (CNS) requires a continuous, tightly controlled supply of oxygen and nutrients as well as washout of harmful metabolites through uninterrupted cerebral blood flow (CBF). The energetic demands of neurons are very high, yet the brain has very little energetic reserves. During periods of intense neuronal activity, there is a requirement for adjusting oxygen and glucose delivery to local neuronal activity through rapid adaptive increases in CBF. This is ensured by a mechanism known as neurovascular coupling (NVC). The resultant functional hyperemia is a vital mechanism to maintain optimal microenvironment of cerebral tissue and thereby ensuring normal neuronal function.

There is an increasing appreciation that (micro)vascular contributions to cognitive impairment and dementia in elderly patients are critical (Tarantini, Tran, Gordon, Ungvari & Csiszar, 2016). Importantly, neurovascular coupling responses are impaired both in elderly patients (Fabiani et al., 2013; Stefanova et al., 2013; Topcuoglu, Aydin & Saka, 2009; Zaletel, Strucl, Pretnar‐Oblak & Zvan, 2005) and aged laboratory animals (Park, Anrather, Girouard, Zhou & Iadecola, 2007; Toth et al., 2014), which likely contributes significantly to the progressive age‐related decline in higher cortical function, including cognition (Sorond, Hurwitz, Salat, Greve & Fisher, 2013) and gait coordination (Sorond et al., 2011). Experimental studies support this concept, showing that pharmacologically induced neurovascular uncoupling in mice mimics important aspects of age‐related cognitive impairment (Tarantini et al., 2015). On the basis of these findings, we proposed that novel therapeutic interventions should be developed to rescue functional hyperemia in elderly patients to prevent/delay cognitive impairment (Toth et al., 2014).

There is strong evidence that microvascular endothelial NO synthesis/release is stimulated during increased neuronal activity (via ATP‐mediated activation of endothelial purinergic receptors), which critically contribute to functional hyperemia under normal conditions (Toth, Tarantini, Davila et al., 2015). Previous studies demonstrate that aging exacerbates generation of reactive oxygen species (ROS) in the cerebromicrovascular endothelial cells, which contribute to age‐related neurovascular uncoupling in aged mice by promoting endothelial dysfunction (Toth et al., 2014). We hypothesize that pharmacological treatments, which attenuate endothelial oxidative stress and rescue NO mediation, will have the capacity to improve neurovascular coupling in aged individuals.

The mitochondrial free radical theory of aging posits that mitochondria‐derived ROS (mtROS) production and related mitochondrial dysfunction are a critical driving force in the aging process. In support of this theory, it was demonstrated that attenuation of mitochondrial oxidative stress (by mitochondria‐targeted overexpression of catalase) increases mouse lifespan (Schriner & Linford, 2006). Further, pharmacological treatments that attenuate mtROS production were shown to counteract some of the effects of aging in skeletal muscle (Siegel et al., 2013) and the heart (Dai, Chiao, Marcinek, Szeto & Rabinovitch, 2014; Dai, Rabinovitch & Ungvari, 2012; Dai et al., 2011). There is particularly strong evidence that mitochondrial oxidative stress is implicated in cardiovascular aging processes (Dai et al., 2012; Springo et al., 2015). Yet, although drugs that improve mitochondrial function have been shown to exert beneficial effects both on the vasomotor function of peripheral arteries (Gioscia‐Ryan et al., 2014; Pearson et al., 2008), their potential protective effects on the aged cerebral microvasculature has not been investigated.

This study was designed to test the hypothesis that pharmacological attenuation of mtROS can restore cerebromicrovascular endothelial function and thus improve neurovascular coupling in aged mice. To achieve this goal, in aged mice mitochondrial oxidative stress was manipulated by treatment with the mitochondrial‐targeted Szeto‐Schiller (SS) peptide SS–31. SS–31 is a tetrapeptide with alternating aromatic‐cationic amino acid motif (H‐D‐Arg‐Dmt‐Lys‐Phe‐NH_2_), which concentrates in the inner mitochondrial membrane more than 1,000‐fold compared with the cytosolic concentration (Bakeeva et al., 2008; Doughan & Dikalov, 2007; Zhao et al., 2004) and is able to effectively reduce levels of O_2_^−^, H_2_O_2,_ hydroxyl radical and peroxynitrite both in vitro and in vivo (Graham et al., 2009; Szeto, 2008; Zhao et al., 2004). Mice were behaviorally evaluated on a battery of tests for characterization of cognitive function and motor coordination and functional tests for neurovascular coupling responses and cerebromicrovascular endothelial function. Markers of oxidative stress and expression of genes regulating neurovascular coupling responses in the cerebral cortex were assessed. To substantiate the in vivo findings, the effects of SS–31 on mitochondrial ROS production and mitochondrial respiration in cerebromicrovascular endothelial cells derived from aged animals were obtained in vitro.

## 2 RESULTS

### 2.1 Treatment with SS‐31 rescues neurovascular coupling responses in aged mice by restoring NO mediation

**Figure 1:**
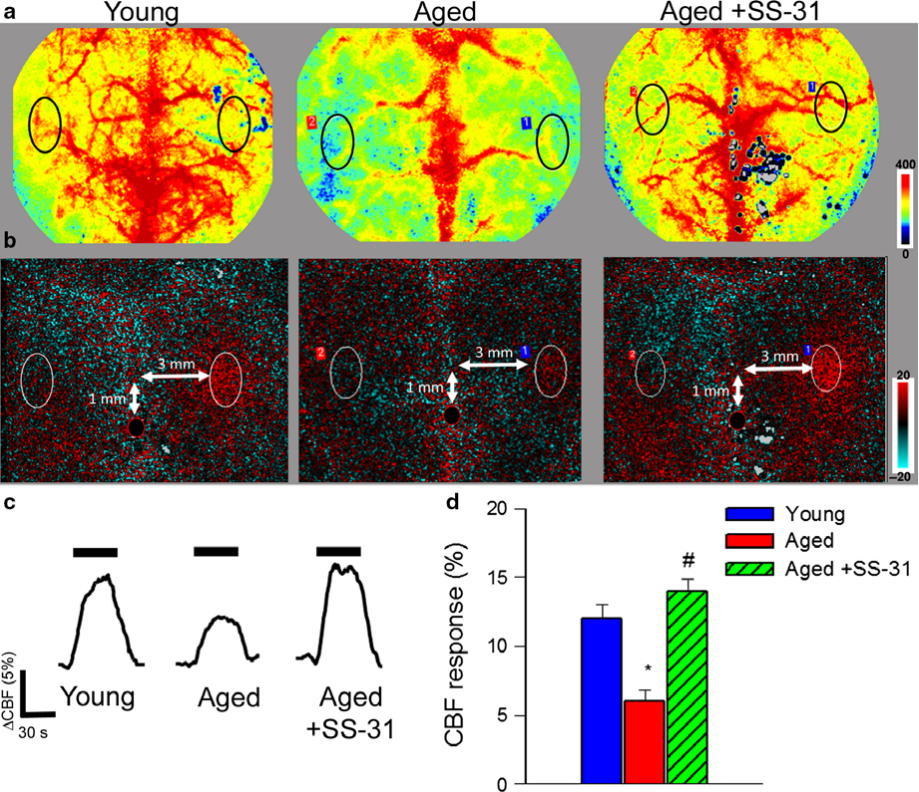
Treatment with SS‐31 rescues neurovascular coupling in aged mice. Panel a–b: Representative pseudocolor laser speckle flowmetry maps of baseline CBF (Panel a) and CBF changes in the whisker barrel field relative to baseline during contralateral whisker stimulation (Panel b, right oval, 30 s, 5 Hz) in young (3 months old), aged (24 months old), and SS‐31‐treated aged mice. Color bar represents CBF as percent change from baseline. Panel c shows the time course of CBF changes after the start of contralateral whisker stimulation (horizontal bars). Summary data are shown in panel d. Data are mean ± *SEM*. (*n* = 6–8 in each group), *p < .05 vs. young; ^#^ *p* < .05 vs. aged. (one‐way ANOVA with post hoc Tukey's tests)

CBF responses in the whisker barrel cortex elicited by contralateral whisker stimulation were significantly decreased in aged mice compared to young animals indicating impaired neurovascular coupling in aging (representative laser speckle contrast images and CBF tracings are shown in Figure 1a,c; summary data are shown in Figure 1b,d)(Park et al., 2007). We found that a 14‐day treatment with SS–31 significantly increased CBF responses induced by contralateral whisker stimulation in aged mice, restoring neurovascular coupling to levels observed in young mice (Figure 1).

**Figure 2:**
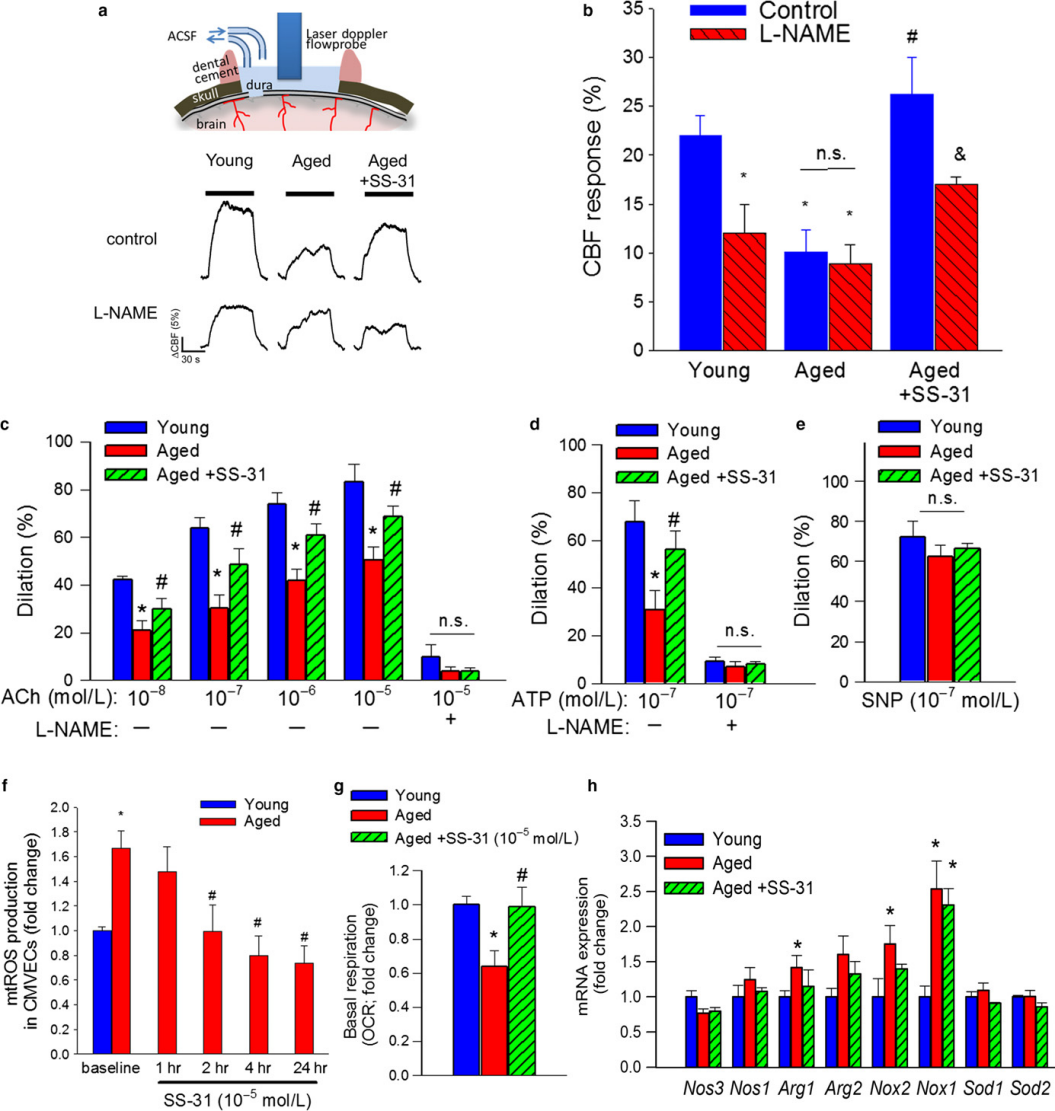
Treatment with SS‐31 improves microvascular endothelial function and rescues NO mediation of neurovascular coupling response in aged mice. Panel a: Representative traces of cerebral blood flow (CBF; measured with a laser Doppler probe above the whisker barrel cortex) during contralateral whisker stimulation (30 s, 5 Hz) in the absence and presence of the NO synthase inhibitor L‐NAME in young (3 months old), aged (24 months old), and SS‐31‐treated aged mice. Summary data are shown in panel b. *p < .05 vs. young; ^#^ *p* < .05 vs. aged. (one‐way ANOVA with post hoc Tukey's tests) Panel c–d: Dilation of cannulated branches of the middle cerebral artery, isolated from young, aged, and SS‐31‐treated aged mice, in response to the endothelium‐dependent vasodilator acetylcholine (c) and ATP (d) in the absence and presence of the NO synthase inhibitor L‐NAME (3 × 10^−4^ M). Panel e: Vascular dilations to the endothelium‐independent aged.; n.s.: not significant. Panel f: Time course for changes in mtROS production in aged CMVECs induced by treatment with SS‐31 (10^−5^ M). Data are mean ± *SEM*. (*n* = 8 in each group). *p < .05 vs. young control; ^#^ *p* < .05 vs. aged control. Panel g: Attenuation of mtROS production in aged CMVECs was associated with significant improvement in cellular oxygen consumption rate (OCR; a marker of oxidative phosphorylation; see Methods). Data are mean ± *SEM*. (*n* = 8 in each group). *p < .05 vs. young control; ^#^ *p* < .05 vs. aged control. Panel h: qPCR data showing cortical mRNA expression of nitric oxide synthases *Nos3* and *Nos1*, arginases (*Arg1, Arg2*), NADPH oxidases (*Nox1, Nox2*), and superoxide dismutases (*Sod1, Sod2*) in young, aged, and SS‐31‐treated aged mice. Data are mean ± *SEM*. (*n* = 6–7 in each group). *p < .05 vs. young control; ^#^ *p* < .05 vs. aged control

Studies using an open cranial window preparation showed that in young animals administration of the NO synthase inhibitor L‐NAME significantly decreased neurovascular coupling responses, eliminating the differences between the age groups (representative laser Doppler tracings are shown in Figure 2a; summary data are shown in Figure 2b). In untreated aged animals, administration of L‐NAME was without effect (Figure 2a,b). In contrast, in SS–31‐treated aged mice L‐NAME significantly decreased CBF responses elicited by whisker stimulation (Figure 2a,b), suggesting that SS–31 treatment restored the NO mediation of neurovascular coupling in aged animals.

### 2.2 Treatment with SS‐31 improves endothelial function in aged cerebral vessels

To further ascertain the endothelial protective effects of SS‐31, endothelium‐dependent vasodilator responses were tested in isolated, cannulated branches of the MCA. Pressurized MCAs developed spontaneous myogenic tone (~30%), the magnitude of which did not differ between the experimental groups. In young vessels, administration of acetylcholine (Figure 2c) and ATP (Figure 2d) resulted in significant dilation, whereas these responses were significantly attenuated in vessels derived from aging mice (Figure 2c,d). Treatment of aged mice with SS‐31 significantly improved both acetylcholine‐ and ATP‐induced vasodilation, restoring responses to the level observed in vessels of young mice. To assess the role of endothelium‐derived NO, L‐NAME was applied. L‐NAME significantly inhibited acetylcholine‐ and ATP‐induced dilator responses, eliminating the differences between the three groups. These finding suggests that SS‐31 significantly improves endothelial function by restoring endothelial NO mediation in aged cerebral vessels. Vasodilator responses elicited by administration of the NO donor sodium nitroprusside (SNP) did not differ significantly among the experimental groups (Figure 2e), suggesting that the dilator capacity of smooth muscle cells in the aged cerebral vasculature is preserved and is unaffected by SS‐31.

### 2.3 SS‐31 attenuates mitochondrial oxidative stress and improves mitochondrial respiration in aged cerebromicrovascular endothelial cells

To substantiate the endothelial protective effects of SS‐31 in vitro, we assessed the effects of SS‐31 on cellular mtROS production in cultured CMVECs derived from aged animals using the MitoSox fluorescence method. We found that mtROS production in CMVECs derived from aged animals was significantly increased as compared to that in CMVECs derived from young animals (Figure 2f). SS‐31 treatment elicited significant decreases in mtROS production in aged CMVECs, eliminating the difference between the two age groups (Figure 2f). The antioxidative effects of SS‐31 were manifested rapidly, with maximal reduction in mtROS being evident after 2 hr post‐treatment (Figure 2f). Attenuation of mtROS production in aged CMVECs was associated with significant improvement in mitochondrial respiration (Figure 2 g), which is compatible with the idea that SS‐31 energizes mitochondria, promoting electron transport and optimizing mitochondrial ATP synthesis at the same time as it reduces ROS generation by escape of free electrons from the respiratory chain (Birk, Chao, Bracken, Warren & Szeto, 2014; Birk et al., 2013).

### 2.4 Effects of treatment with SS‐31 on expression of genes regulating NO mediation in the aged mouse brain

Treatment with SS‐31 did not alter the expression of *Nos1* and *Nos3* in the aged mouse brain (Figure 2h). The expression of arginase 1 was increased in aged mice and tended to be downregulated by SS‐31 treatment. NADPH oxidases are also important sources of ROS in the cerebral vasculature, whose increased expression and activity may contribute to impaired neurovascular coupling (Park et al., 2007; Toth et al., 2014). We found that in the cortex of aged mice mRNA expression of the NADPH‐oxidase subunits *Nox1* and *Nox2* were upregulated compared to young mice (Figure 2i), extending recent findings (Park et al., 2007; Toth et al., 2014). Expression of *Nox2* in SS‐31‐treated aged CMVECs was statistically not different from that in young CMVECs (Figure 2h). Treatment with SS‐31 did not alter the expression of *Nox1, Sod1* and *Sod2* in the aged mouse brain (Figure 2h).

### 2.5 Restoration of cerebromicrovascular function is associated with improved cognitive function in aged mice treated with SS‐31

**Figure 3:**
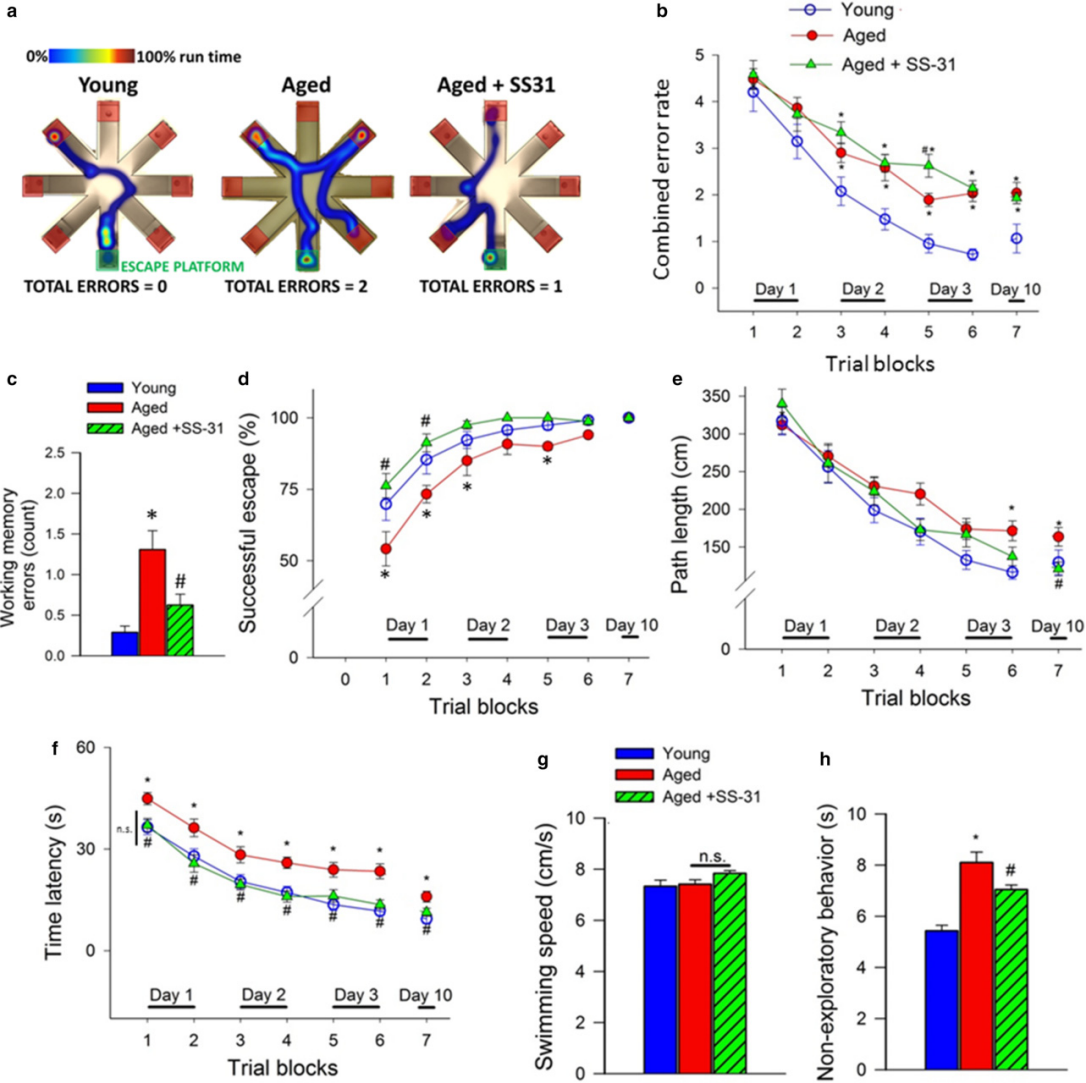
In SS‐31‐treated aged mice, rescue of neurovascular coupling responses associates with improved radial‐arm water maze (RAWM) performance. Young (3 months old), aged (24 months old), and SS‐31‐treated aged mice were tested in the RAWM. Panel a: Heatmap representing the percentage of time spent in different locations in the maze for a randomly selected animal from each group during experimental day 3. Note that the untreated aged mouse required a greater amount of time and a longer path length in order to find the hidden escape platform. Older mice also re‐enter a previously visited arm multiple time, accruing working memory errors. Panel b: Older animals have higher combined error rates throughout days 2 and 3 of the learning phase and retrieval day 10 (*p* < .0001). Combined error rate is calculated by adding 1 error for each incorrect arm entry as well as for every 15 s spent not exploring the arms. Panel c: Older animals make significantly more working memory errors (repetitive incorrect arm entries) as compared to young mice. In contrast, aged mice treated with SS‐31 perform this task significantly better than untreated aged mice (*p* = .04). Panel d: The ratio of successful escapes, averaged across trial blocks, is shown for each group. Note day‐to‐day improvement in the performance of young mice, which was significantly delayed in aged mice. Aged mice treated with SS‐31 were more successful at finding the hidden escape platform in comparison with untreated age‐matched controls (*p* < .0001). Panel e: Average path length required to reach the hidden platform in the RAWM for trial blocks 1–6 and for probe trial on day 10. Young mice find the hidden platform while swimming significantly less than aged animals (*p* < .0001). In aged mice treated with SS‐31, the average path length required to reach the hidden platform did not differ from that in young mice. Panel f: Escape latencies to find the hidden platform in the RAWM for aged control mice are significantly higher when compared to young and aged treated mice (*p* < .0001 for both comparisons). Panel g: There was no significant difference in swimming speed among all experimental groups. Panel h: SS‐31 treatment reduced the time spent engaged in nonexploratory behavior in control‐treated old mice as compared to young animals. (*p* < .0001). *n* = 20 in each group. All data are shown as mean ± *SEM*. Statistical significance was calculated using one‐way ANOVA with Tukey's post hoc test to determine differences among groups

Previous studies show that selective pharmacologically induced neurovascular uncoupling is associated with cognitive impairment (Tarantini et al., 2015). To determine how restoration of cerebromicrovascular function impacts cognitive function in aged mice, animals were tested in the radial‐arm water maze (Figure 3a). We compared the learning performance of mice in each experimental group by analyzing the day‐to‐day changes in the combined error rate, successful escape rate, degree of divergence, path length, and time latency. During acquisition, mice from all groups showed a decrease in the combined error rate (Figure 3b) across days, indicating learning of the task. After the first day of learning, young mice consistently had lower combined error rate than aged mice (Figure 3b). Treatment with SS‐31 did not alter the combined error rate in aged mice.

To analyze working memory function (short‐term memory that is involved in immediate conscious perception), we examined re‐entries into incorrect arms (without hidden platform) that were previously attempted for escape. We found that working memory function was impaired in aged mice as compared to young controls (Figure 3c). Aged mice that were treated with SS‐31 showed significant restoration of working memory to levels comparable to young animals (Figure 3c). SS‐31 treatment thus resulted in a marked improvement in working memory while there was no clear effect of SS‐31 on learning.

Successful escape rate from the maze was assessed by measuring the percent of animals that could find the hidden platform within the 60 s allowed for each trial. During acquisition, mice from all groups showed an increase in successful escape rate consistent with the learning of the task. Young mice exhibited significantly better escape success than untreated aged mice (Figure 3d). SS‐31 treatment significantly increased the successful escape rate in aged mice, restoring it to the level observed in young mice (Figure 3d). We attribute, at least in part, the higher successful escape rate in young maze‐naïve mice and SS‐31‐treated aged maze‐naïve mice to the more effective search strategy they used as indicated by the lower degree of divergence.

We also compared path length (i.e., the distance that the mouse swam between maze entry and successful escape through the hidden platform) and escape latency (i.e., the time elapsed between entry and successful escape). During acquisition, mice from all groups displayed shorter path length (Figure 3e) and lower escape latencies (Figure 3f), indicating spatial learning. Young mice exhibited significantly shorter path length (Figure 3e) and lower escape latency (Figure 3f) than untreated aged mice, which differences remained pronounced on day 3 of acquisition and the probe trial. In contrast, in aged mice treated with SS‐31 path length and escape latencies were similar to that observed in young mice (Figure 3e,f). The analyses of noncognitive parameters revealed no difference in swimming speed and only a slight, age‐dependent increase in nonexploratory behavior (the cumulative time the mice spent not actively looking for the platform, e.g., floating), which was reduced in SS‐31‐treated animals (Figure 3g,h). Taken together, the aforementioned results suggest that in aged mice SS‐31 treatment improved performance in the radial‐arm water maze, which results from enhanced hippocampal‐dependent spatial learning and memory and not from changes in motor or motivational processes.

### 2.6 Treatment with SS‐31 restores motor learning function in aged mice

To investigate the effects of age and SS‐31 treatment on the motor performance of mice, we measured grip strength and inverted grid hang time (data not shown), which evaluates muscle strength and endurance. We found that neither aging nor SS‐31 treatment affected the outcome of these tests.

**Figure 4:**
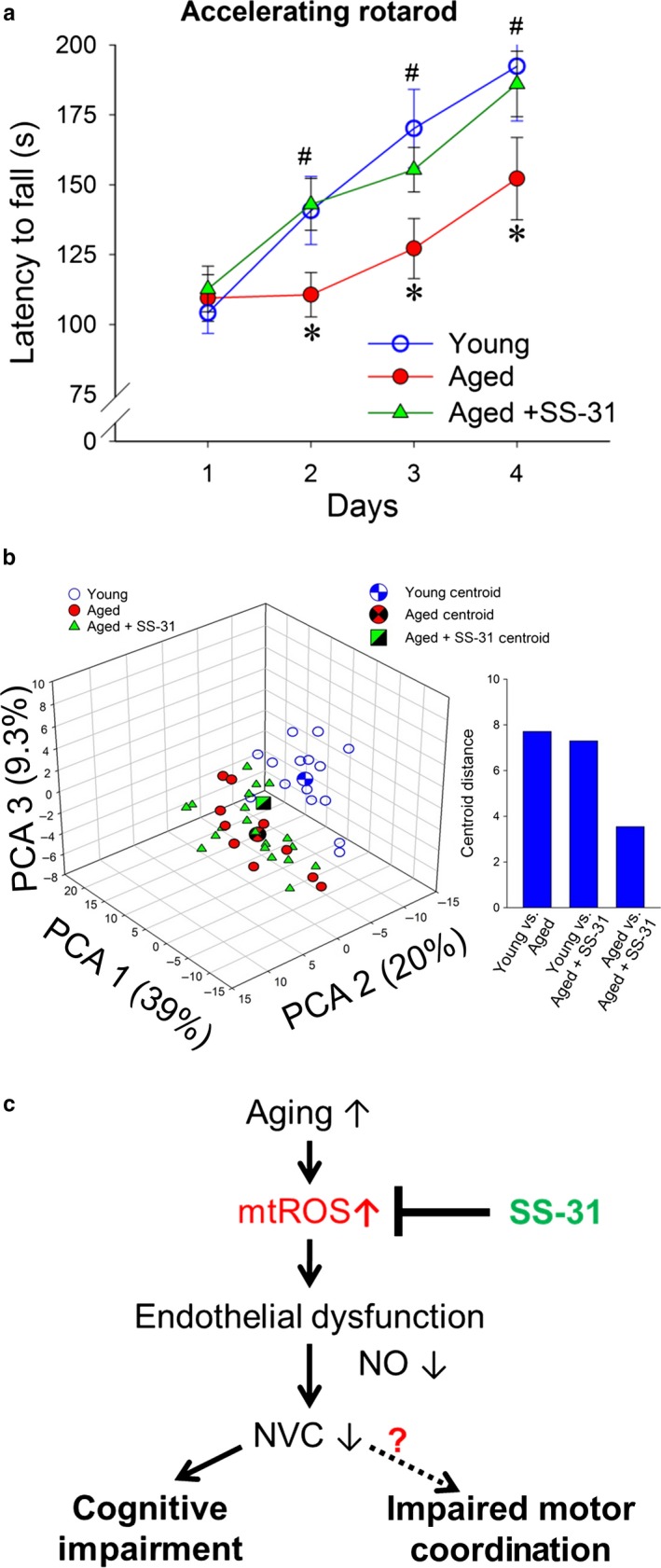
SS‐31 treatment in aged mice improves motor skill learning but not gait coordination. a) Motor skill learning on the accelerating rotarod. Daily changes in mean latencies to fall for young (3 months old), aged (24 months old) and SS‐31‐treated aged mice are shown. Data are mean ± *SEM*. (*n* = 20 in each group). *p < .05 vs. young control; ^#^ *p* < .05 vs. aged control. b) 3D triplot of first three principal components (PC) identified by PCA on the correlation matrix of spatial and temporal indices of gait. Each point represents an individual mouse, and centroids are shown for each experimental group. Note that mice in the same age groups clustered together. Distance between centroids is shown inset. Whole differences between young and aged mouse gait were evident, rescue of NVC responses by SS‐31 treatment did not reverse age‐related changes in mouse gait (MANOVA;* p* = .47 aged vs. aged treated; *p* < .001 young vs. aged; *p* < .0001 young vs. aged treated). c) Scheme showing proposed role for increased mitochondrial oxidative stress in cerebromicrovascular endothelial impairment and neurovascular dysfunction in aging

Motor skill learning was assessed using a modified rotarod test. During a 4‐day‐long experimental period, young mice displayed a steeper motor skill learning curve compared to aged control mice. After the first day, aged control animals consistently fell off the accelerating rotarod earlier compared to young controls, showing impaired acquisition of motor skill learning. Treatment with SS‐31 restored motor skill learning behavior to the young control levels (Figure 4a).

### 2.7 Treatment with SS‐31 does not restore gait function in aged mice

Age‐related deficiencies in neurovascular coupling responses in human patients have been linked to gait abnormalities (Sorond et al., 2011). To identify subtle age‐ and treatment‐related differences between mouse gait patterns, principal component analysis (PCA) was carried out on the correlation matrix of averages of spatial and temporal indices of gait. This analysis identified three principal components that accounted for ~68% of the variance in the data. We plotted the position of each mouse against the PC1, PC2, and PC3 axes in three‐dimensional space (Figure 4b). The most conspicuous trend was that young and aged mice appeared well separated along the PC2 and PC3 axes. In contrast, aged mice and SS‐31‐treated aged mice were clustered together.

## 3 DISCUSSION

The key finding of his study is that the mitochondria‐targeted antioxidative peptide SS‐31 exerts significant beneficial effects on neurovascular coupling responses and cognitive function in a mouse model of aging that recapitulates cerebromicrovascular dysfunction and deficits of higher brain function manifested in elderly patients.

Extending previous findings in elderly patients and aged laboratory animals, we demonstrate that aging leads to significant neurovascular dysregulation in mice, characterized by diminished CBF changes in response to increased neuronal activity (Figure 1). Here, we demonstrate for the first time that aging‐induced decline in neurovascular coupling is restored by treatment with SS‐31 (Figure 1). There is strong experimental evidence that endothelial NO production contributes importantly to neurovascular coupling (Toth et al., 2014). This concept is supported by our observation that both genetic depletion of eNOS (Toth, Tarantini, Davila et al., 2015) and pharmacological inhibition of NO synthesis (Figure 2a,b) dramatically reduce neurovascular coupling in young mice. The findings that in aged mice inhibition of endothelial NO synthesis does not attenuate functional hyperemia (Toth et al., 2014) (Figure 2a) suggest that cerebromicrovascular endothelial dysfunction significantly contributes to age‐related neurovascular uncoupling (Figure 2b)(Park et al., 2007). Importantly, treatment with SS‐31 rescues NO mediation of neurovascular coupling in aged animals (Figure 2a) supporting the concept that potent endothelial protective effects of SS‐31 play a key role in its anti‐aging action. Additional evidence in support of this concept comes from the observations that SS‐31 treatment rescued acetylcholine‐ and ATP‐induced, endothelial NO‐mediated vasodilation in aged mice (Figure 2c,d). It should be noted that endothelium‐derived NO also modulates cellular metabolism and mitochondrial function, is an important inhibitor of platelet aggregation, smooth muscle cell proliferation and leukocyte adhesion, and exerts potent anti‐inflammatory, anti‐apoptotic, and pro‐angiogenic effects. Therefore, SS‐31 mediated improvement in microvascular NO production likely has clinical significance beyond rescue of NVC response, potentially exerting multifaceted protective effects on neuronal, astrocytic and microglial functions.

Previous studies demonstrate that the mechanism(s) by which aging impairs cerebromicrovascular endothelial function involves an increased breakdown of NO by elevated levels of ROS (Toth et al., 2014). On the basis of the findings that SS‐31 treatment effectively attenuates oxidative stress in aged CMVECs (Figure 2f–g), we propose that in aged mice SS‐31 treatment restores NVC responses and endothelium‐dependent cerebromicrovascular dilation by preventing oxidative stress‐mediated scavenging of NO in the microvascular endothelial cells. Recovery of endothelial function in aged peripheral arteries has also been reported using the structurally different mitochondria‐targeted antioxidant MitoQ (Gioscia‐Ryan et al., 2014).

The mechanisms by which SS‐31 attenuates mtROS production in aging are likely multifaceted. Age‐related loss of efficiency in mitochondrial electron transport likely increases electron leak and mtROS production. SS‐31 is known to concentrate in the inner mitochondrial membrane more than 1,000‐fold compared with the cytosolic concentration (Bakeeva et al., 2008; Doughan & Dikalov, 2007; Zhao et al., 2004). While SS‐31 may scavenge ROS directly at the site of their production, recent studies show that SS‐31 binds to cardiolipin via electrostatic and hydrophobic interactions and thereby protects the structure of mitochondrial cristae, increasing the efficiency of mitochondrial electron transport (Siegel et al., 2013) and attenuating mtROS production (Szeto, 2014). Increased efficiency of ATP generation and reduction in ROS are thereby coupled. Based on the findings that SS‐31 improves mitochondrial respiration in cerebromicrovascular endothelial cells (Figure 2h), while reducing ROS generation (Figure 2f,g) we posit that such a mechanism is operational in the cerebral microcirculation of SS‐31‐treated aged mice. There are studies suggesting that upregulation of the tissue renin–angiotensin II system (Wang et al., 2007) contributes to increased vascular oxidative stress in aging (Modrick, Didion, Sigmund & Faraci, 2009). Importantly, SS‐31 was also shown to effectively attenuate angiotensin II‐induced mtROS production (Dai et al., 2011). The microvascular endothelium in the brain, which maintains the blood–brain barrier and exhibits controlled transcellular transport systems, has high energy demands. Future studies should determine how SS‐31‐induced changes in cellular energetics impact barrier and transport function in aged CMVECs. In addition, there is growing evidence that mitochondria‐derived ROS regulate the expression and activity of plasma membrane‐associated NADPH oxidases in vascular cells (reviewed in Dai et al. (2012)). Accordingly, in the cerebral circulation of aged mice increased mitochondrial oxidative stress is associated with an upregulated expression and activity of NADPH oxidases (Park et al., 2007; Toth et al., 2014), which appear to be partially reversed by SS‐31 (Figure 2i) as well as by other interventions that attenuate mtROS production in aging (Toth et al., 2014). Interestingly, SS‐31‐induced downregulation of NADPH oxidases has also been observed previously in the heart (Dai et al., 2011) and the kidney (Hou et al., 2016). Thus, we posit that SS‐31 may also alter the interplay between mitochondria and NADPH oxidases, disrupting a vicious feed‐forward cycle that diminishes NO bioavailability and promotes neurovascular uncoupling. Furthermore, one can hypothesize that SS‐31 treatment of aged mice may also attenuate astrocyte‐derived ROS generation, in addition to its beneficial endothelial effects, thereby improving astrocyte‐mediated neurovascular coupling responses (e.g., by decreasing production of constrictor arachidonic acid metabolites). This possibility should be tested in future studies.

There is a growing appreciation of vascular contributions to cognitive decline and dementia. Importantly, clinical studies suggest that impairment of NVC responses contributes to the age‐related decline in higher cortical functions (Sorond et al., 2011, 2013). Recent studies demonstrate that BOLD signal is positively correlated with working memory in rodent models and that impaired memory is associated with impaired NVC (reduced BOLD activity) in the hippocampus (Febo & Foster, 2016). Our recent studies provide strong experimental evidence in support of this concept, demonstrating that impairment of NVC responses and cognitive decline in mice are causally related (Tarantini et al., 2015). The present study is the first to demonstrate that restoration of NVC by SS‐31 treatment in aging is associated with improvement in multiple domains of brain function, including hippocampal‐dependent memory functions (Figure 3). Previously, we found that restoration of NVC responses in aged mice by treatment with resveratrol (Toth et al., 2014) is also associated with marked improvement in cognitive function (Oomen et al., 2009). It is significant that resveratrol is a potent inhibitor of mtROS production in endothelial cells (Ungvari et al., 2009). Human studies show that procedural motor learning is affected by NVC responses (Grafton, Woods & Tyszka, 1994) and becomes significantly impaired during aging. It is thus significant that SS‐31 treatment rescues motor skill learning in aged mice (Figure 4). Age‐related deficiencies in NVC responses in human patients have been also linked to gait abnormalities (Sorond et al., 2011). Recent studies also show that in a mouse model of radiation‐induced accelerated brain aging (Ungvari et al., 2017) impaired NVC also predicts gait abnormalities. The present study identified systematic differences between young and aged mouse gait using PCA. However, rescue of NVC responses by SS‐31 treatment did not reverse age‐related changes in mouse gait (Figure 4c).

Our findings have important clinical relevance. In humans, SS‐31 (elamipretide) is well tolerated with no reports of significant side effects (Szeto, 2014) and its potential to exert renoprotective and cardioprotective effects is currently being evaluated in clinical trials (Szeto, 2014). Thus, future clinical trials with SS‐31 in elderly patients are feasible, which would allow its potential in improving cerebromicrovascular and cognitive outcomes to be evaluated. Importantly, NVC is also compromised both in patients with Alzheimer's disease (AD) and in animal models of AD, which is believed to trigger and/or exacerbate cognitive decline (Tarantini et al., 2016). Further, the mitochondria‐targeted antioxidant MitoQ was shown to prevent cognitive decline in mouse models of AD (McManus, Murphy & Franklin, 2011). Thus, our results are also likely relevant to the development of novel treatments of AD as well. Importantly, SS‐31 and related tetrapetides can cross the blood–brain barrier; thus, long‐term treatment has the added advantage that it may inhibit inflammatory processes associated with AD as well.

In conclusion, our results demonstrate that SS‐31 treatment exerts cerebromicrovascular protective effects in aged mice. SS‐31 treatment improved endothelial function and rescued neurovascular coupling responses in the aged cortex, which likely contributes to improvements of higher cortical function. Our findings point to the potential use of mitochondria‐targeted antioxidants as therapy for prevention of vascular cognitive impairment associated with aging.

## 4 EXPERIMENTAL PROCEDURES

Materials, experimental procedures, and statistical analyses are described in detail in the Data S1.

### 4.1 Animals, SS‐31 treatment

Young (3 months, *n* = 30) and aged (24 months, *n* = 40) male C57BL/6 mice were used. Mice in the aged cohort were assigned to two groups (*n* = 20 each group). One group of the aged mice was injected daily with SS‐31 (elamipretide; also known as MTP‐131 or Bendavia, 10 mg Kg^−1^day^−1^, for 10 days). This dosage of SS‐31 has been shown to exert potent cardioprotective effects (Dai et al., 2011). All procedures were approved by the Institutional Animal Use and Care Committees of the University of Oklahoma Health Sciences Center.

### 4.2 Behavioral studies

After the treatment period, behavioral tasks were performed to characterize the effect of SS‐31 treatment on learning and memory, sensory‐motor function, gait and locomotion (*n* = 20 in each group).

### 4.3 Radial‐arm water maze testing

Spatial memory and long‐term memory in each group of mice was tested using the radial‐arm water maze as described (Ungvari et al., 2017).

### 4.4 Rotarod, motor skill learning

Motor coordination was assessed using the rotarod as described (Tarantini et al., 2015). Analysis of day‐to‐day changes in performance on the accelerating rotarod test was used to evaluate the motor skill learning. The grid hanging test was also used to evaluate balance and motor function. A grip strength test was used to measure the maximal muscle strength of forelimbs of the mice.

### 4.5 Analysis of gait function

To determine how aging and SS‐31 treatment affect gait coordination, we tested the animals using an automated computer‐assisted method (CatWalk; Noldus Information Technology Inc. Leesburg, VA, USA) as described (Tarantini et al., 2015; Toth, Tarantini, Springo et al., 2015).

### 4.6 Measurement of neurovascular coupling responses

After behavioral testing, mice in each group were anesthetized with isoflurane (4% induction and 1% maintenance), endotracheally intubated, and ventilated. Neurovascular coupling responses were assessed by measuring changes in cerebral blood flow above the left barrel cortex using laser speckle contrast imaging in response to contralateral whisker stimulation (Toth et al., 2014; Toth, Tarantini, Ashpole et al., 2015; Toth, Tarantini, Davila et al., 2015). In a separate cohort of mice, the role of NO mediation in NVC responses was tested. In brief, in anesthetized, intubated and ventilated mice equipped with an open cranial window, changes in CBF were assessed above the left barrel cortex using a laser Doppler probe in response to electric stimulation of the contralateral whisker pad, as described (Tarantini et al., 2015; Toth et al., 2014; Toth, Tarantini, Ashpole et al., 2015). Changes in CBF were averaged and expressed as percent (%) increase from the baseline value. To assess the role of NO mediation, CBF responses to whisker stimulation were repeated in the presence of the nitric oxide synthase inhibitor N^ω^‐Nitro‐L‐arginine methyl ester (L‐NAME; 3 × 10^−4^ M, 20 min).

### 4.7 Assessment of endothelial NO‐mediated vasodilation in isolated cerebral vessels

To assess the specific effect of SS‐31 treatment on endothelial NO mediation, isolated segments of the middle cerebral arteries were cannulated and pressurized, as reported (Springo et al., 2015; Toth et al., 2013). Dilations to acetylcholine (10^−8^–10^−5^ M) and ATP (10^−7^ M) (administered in the bath) were obtained in the absence and presence of L‐NAME (3 × 10^−4^ M, for 30 min). To assess endothelium‐independent vasodilation, responses to the NO donor sodium nitroprusside (SNP, 10^−7^ M) were assessed. At the end of each experiment, the vessels were superfused with Ca^2+^‐free Krebs’ buffer containing nifedipine (10^−5^ M) to achieve maximal vasodilatation.

### 4.8 Quantitative real‐time RT–PCR

A quantitative real‐time RT–PCR technique was used to analyze mRNA expression in cortical samples using validated TaqMan probes (Applied Biosystems) and a Strategen MX3000 platform, as previously reported (Toth, Tarantini, Ashpole et al., 2015).

### 4.9 Assessment of the effect of in vitro treatment with SS‐31 on age‐related increases in mtROS production in cultured cerebromicrovascular endothelial cells

To confirm the direct antioxidative endothelium‐protective effect of SS‐31 in vitro, we assessed the effect of SS‐31 (10^−9^–10^−5^ M, for 24 hr or 10^−5^ M, for 1–24 hr) on mtROS production in cultured primary cerebromicrovascular endothelial cells (CMVECs) derived from young and aged rats. The establishment and characterization of the cell strains used have been recently reported (Csiszar et al., 2014). Mitochondrial O_2_^−^ production in CMVECs was measured by flow cytometry using MitoSox Red (3 μM for 30 min), a mitochondrion‐specific hydroethidine‐derivative fluorescent dye, as reported.

### 4.10 Seahorse respirometry

To substantiate the endothelium‐protective effect of SS‐31, we performed real‐time measurements of the oxygen consumption rate (OCR; a marker of oxidative phosphorylation) in young and aged CMVECs after treatment with SS‐31 (10^−5^ M, for 48 hr) using a Seahorse XF96 extracellular flux analyzer.

### 4.11 Statistical analysis

Data were analyzed by one‐way analysis of variance (ANOVA) followed by Tukey's post hoc test. Principal component analysis, followed by MANOVA and the PCA‐biplot approach were used to analyze the gait data. A *p* value less than .05 was considered statistically significant. Data are expressed as mean ± *SEM*.

## CONFLICT OF INTEREST

None.

## AUTHOR CONTRIBUTION

ST, AY, EF, AC, and ZU designed research; MNVA, ST, AY, GAF, PH, and TG performed experiments; ST, MNVA, AY, GAF, PH, EF, AP, AC, and ZU analyzed data; ST, AC, and ZU wrote the paper. AY, WES, PR, and EF revised the paper.
