# Supplementary material for: Treatment with the mitochondrial‐targeted antioxidant peptide SS‐31 rescues neurovascular coupling responses and cerebrovascular endothelial function and improves cognition in aged mice
Source: Aging Cell. 2018 Feb 6;17(2):e12731. doi: 10.1111/acel.12731 (PMC5847870; doi:10.1111/acel.12731)
Supplement: Supplementary file 1 [file ACEL-17-e12731-s001.docx]

**Treatment with the mitochondrial-targeted antioxidant peptide SS-31 rescues neurovascular coupling responses and cerebrovascular endothelial function and improves cognition in aged mice**

Stefano Tarantini, PhD^1,2*^, M Noa Valcarcel-Ares, PhD^1,*^, Andriy Yabluchanskiy, MD, PhD^1,2,*^, Gabor A. Fulop, MD^1,2,*^, Peter Hertelendy,MD^1,3^, Tripti Gautam, MS^1^, Eszter Farkas, PhD^3^, Aleksandra Perz, MS^4^, Peter Rabinovitch, PhD^5^, William E. Sonntag, PhD^1,2^, Anna Csiszar, MD, PhD^1,2,3^, Zoltan Ungvari, MD, PhD^1,2,3^

1) Reynolds Oklahoma Center on Aging, University of Oklahoma Health Sciences Center, Oklahoma City, Oklahoma, USA

2) Translational Geroscience Laboratory, Donald W. Reynolds Department of Geriatric Medicine, University of Oklahoma Health Sciences Center, Oklahoma City, Oklahoma, USA

3) Department of Medical Physics and Informatics, Faculty of Medicine and Faculty of Science and Informatics, University of Szeged, Szeged, Hungary

4) Arthritis & Clinical Immunology Research Program, Oklahoma Medical Research Foundation, Oklahoma City, Oklahoma, USA

5) Department of Pathology, University of Washington, Seattle, Washington, USA

**Online Supplement**

**Detailed Experimental Procedures**

*Animals, SS-31 treatment*

Young (3 month, n=30) and aged (24 month, n=40) male C57BL/6 mice were purchased from the aging colony maintained by the National Institute on Aging at Charles River Laboratories (Wilmington, MA). Animals were housed under specific pathogen-free barrier conditions in the Rodent Barrier Facility at University of Oklahoma Health Sciences Center under a controlled photoperiod (12 h light; 12 h dark) with unlimited access to water and were fed a standard AIN-93G diet (ad libitum). Mice in the aged cohort were assigned to two groups (n=20 each group). One group of the aged mice was injected daily with SS-31 (elamipretide; also known as MTP-131 or Bendavia, 10 mg/Kg/day, for 10 days). This dosage of SS-31 has been shown to exert potent cardioprotective effects([Dai *et al.* 2011](#_ENREF_7)). All procedures were approved by the Institutional Animal Use and Care Committees of the University of Oklahoma Health Sciences Center.

*Behavioral studies*

After the treatment period behavioral tasks were performed to characterize the effect of SS-31 treatment on learning and memory, sensory-motor function, gait and locomotion (n=20 in each group).

*Radial arms water maze testing*

Spatial memory and long term memory was tested by observing and recording escape latency, distance moved, and velocity during the time spend in the radial arms water maze([Shukitt-Hale *et al.* 2004](#_ENREF_12)). The maze consisted of eight arms 9 cm wide that radiated out from an open central area, with a submerged escape platform located at the end of one of the arms. Paint was added into the water to make it opaque. The maze was surrounded by privacy blinds with extramaze visual cues. Intramaze visual cues were placed at the end of the arms. The mice were monitored by a video tracking system directly above the maze as they waded and parameters were measured using Ethovision software Noldus Information Technology Inc., Leesburg, VA, USA). Experimenters were unaware of the experimental conditions of the mice at the time of testing. During the learning period each day, mice were given the opportunity to learn the location of the submerged platform during two sessions each consisting of four consecutive acquisition trials. On each trial, the mouse was started in one arm not containing the platform and allowed to wade for up to one minute to find the escape platform. All mice spent 30 s on the platform following each trial before beginning the next trial. The platform was located in the same arm on each trial. Over the three days of training, mice in the young control group gradually improved performance as they learned the procedural aspects of the task. Upon entering an incorrect arm (all four paws within the distal half of the arm) or failing to select an arm after 15 s the mouse was charged an error. Learning capability was assessed by comparing performance on days 2 and 3 of the learning period. When eventually both aged groups learned the procedural aspects of the task, reaching an asymptotic level of < 2 combined errors on trial block 6, the mice were placed in their home cage for seven day. Then, the mice were administered the retention trial on day 10.

*Rotarod, motor skill learning*

Motor coordination was assessed in each group of mice by using an automated four-lane rotarod (Columbus Instruments, Columbus, OH) as described([Tarantini *et al.* 2015](#_ENREF_14)). Analysis of day-to-day changes in performance on the accelerating rotarod test was used to evaluate the motor skill learning. In brief, mice were pre-trained by placing them on the moving rotarod at 10 r.p.m. until they performed at this speed for 120 sec. On the days of testing, mice were habituated in their home cages and acclimate to the testing room for at least 15 min. The test phase consisted of 3 trials (separated by 15 min inter-trial intervals) per day for 4 days. The testing apparatus was set to accelerate from 4 to 40 r.p.m. in 300 seconds. One mouse was then placed on each lane and the rotarod was started with an initial rotation of 4 r.p.m. The rotational velocity was set to increase every 10 seconds and the latency to fall was recorded. Latency to fall was recorded in seconds by an infra-red beam across the fall path along with the max r.p.m. sustained by each mouse([MacLaren *et al.* 2014](#_ENREF_10)).

*Grid hanging test*

The hanging test was also used to evaluate balance and motor function. In brief, the test began with the mice hanging from an elevated wire grid. The mice were placed on the grid, which was then inverted and suspended above the home cage. The latency to when the animals fell was recorded. The average performance for three trials is reported. Mice that let themselves fall off the grid on purpose were immediately placed back on the grid in the starting position. The test conditions were standardized to minimize variability between sessions.

*Grip strength test*

A grip strength test was used to measure the maximal muscle strength of forelimbs of the mice. Forelimb grip strength was assessed using a grip strength meter (Chatillon Ametek Force Measurement, Brooklyn, New York). The strength measurements of each group of mice were measured three times by the same investigator. The maximum grip strength values were used for subsequent analysis.

*Analysis of gait function*

To determine how aging and SS-31 treatment affect gait coordination, we tested the animals using an automated computer assisted method (CatWalk; Noldus Information Technology Inc. Leesburg, VA) as described([Tarantini *et al.* 2015](#_ENREF_14); [Toth *et al.* 2015b](#_ENREF_16)). Using the CatWalk system the detection of paw print size, pressure and pattern during volunteer running on an illuminated glass walkway by a camera placed under the glass surface provides an automated analysis of gait function and the spatial and temporal aspects of interlimb coordination([Tarantini *et al.* 2015](#_ENREF_14); [Toth *et al.* 2015b](#_ENREF_16)). Briefly, animals were trained to cross the walkway and then, in a dark room and silent room (<20 lux of illumination), animals were tested in three consecutive runs. Data were averaged across ten runs in which the animal maintained a constant speed across the walkway. After manual identification and labeling of each footprint, spatial and temporal indices of gait were calculated (including regularity index, brake and propulsion phase duration, stand index, duty cycle; size-adjusted base of support, stride length and distance between ipsilateral prints; stride length and stride time coefficient of variance, cadence, phase dispersions, walking speed).

*Measurement of neurovascular coupling responses*

After behavioral testing, mice in each group were anesthetized with isoflurane (4% induction and 1% maintenance), endotracheally intubated and ventilated (MousVent G500; Kent Scientific Co, Torrington, CT). A thermostatic heating pad (Kent Scientific Co, Torrington, CT) was used to maintain rectal temperature at 37^o^C ([Toth *et al.* 2014](#_ENREF_17)). End-tidal CO_2_ was controlled between 3.2% and 3.7% to keep blood gas values within the physiological range, as described([Tarantini *et al.* 2015](#_ENREF_14); [Toth *et al.* 2015a](#_ENREF_15)). The right femoral artery was canulated for arterial blood pressure measurement (Living Systems Instrumentations, Burlington, VT) ([Toth *et al.* 2014](#_ENREF_17)). The blood pressure was within the physiological range throughout the experiments (90-110 mmHg). Mice were immobilized and placed on a stereotaxic frame (Leica Microsystems, Buffalo Grove, IL), the scalp and periosteum were pulled aside and the skull was gently thinned using a dental drill while cooled with dripping buffer. A laser speckle contrast imager (Perimed, Järfälla, Sweden) was placed 10 cm above the thinned skull, and to achieve the highest CBF response the right whiskers were stimulated for 30 seconds at 10 Hz from side to side. Differential perfusion maps of the brain surface were captured. Changes in CBF were assessed above the left barrel cortex in six trials in each group, separated by 5-10 min intervals.

In a separate cohort of mice the role of NO mediation in NVC responses was tested. In brief, in anesthetized, intubated and ventilated mice were equipped with an open cranial window and changes in CBF)were assessed above the left barrel cortex using a laser Doppler probe (Transonic Systems Inc., Ithaca, NY), as described([Toth *et al.* 2014](#_ENREF_17); [Tarantini *et al.* 2015](#_ENREF_14); [Toth *et al.* 2015a](#_ENREF_15)). The cranial window was superfused with artificial cerebrospinal fluid (ACSF, composition: NaCl 119 mM, NaHCO_3_ 26.2 mM, KCl 2.5mM, NaH_2_PO_4_ 1mM, MgCl_2_ 1.3mM, glucose 10mM, CaCl_2_ 2.5mM, pH=7.3, 37°C). The right whisker pad was stimulated by a bipolar stimulating electrode placed to the ramus infraorbitalis of the trigeminal nerve and into the masticatory muscles. The stimulation protocol used to investigate neurovascular coupling consisted of 10 stimulation presentation trials with an intertrial interval of 70 seconds, each delivering a 30-second train of electrical pulses (2 Hz, 0.2 mA, intensity, and 0.3 ms pulse width) to the mystacial pad after a 10-second prestimulation baseline period. Changes in CBF were averaged and expressed as percent (%) increase from the baseline value([Kazama *et al.* 2004](#_ENREF_9)). Experiments lasted ~20-30 min/mouse, which permitted stable physiological parameters to be obtained. To assess the role of NO mediation, CBF responses to whisker stimulation were repeated in the presence of the nitric oxide synthase inhibitor N^ω^-Nitro-L-arginine methyl ester (L-NAME; 3x10^-4^ mol/L, 20 min). In each study the experimenter was blinded to the treatment of the animals. At the end of the experiments the animals were transcardially perfused and decapitated. The brains were immediately removed and pieces of the somatosensory and motor cortex were isolated and frozen for subsequent analysis.

*Assessment of endothelial NO-mediated vasodilation in isolated cerebral vessels*

To assess the specific effect of SS-31 treatment on endothelial NO mediation, segments of the middle cerebral arteries (MCA) were isolated using microsurgery instruments, as reported([Springo *et al.* 2015](#_ENREF_13)). In brief, segments of MCAs were mounted onto two glass micropipettes in an organ chamber and pressurized to 60 mmHg. Inner vascular diameter was measured with a custom-built videomicroscope system and continuously recorded using a computerized data acquisition system as reported([Toth *et al.* 2013](#_ENREF_18)). All vessels were allowed to stabilize for 60 min in oxygenated (21% O_2_, 5% CO_2_, 75% N_2_) Krebs’ buffer (at 37°C). Pressurized MCAs developed spontaneous myogenic tone, the magnitude of which did not differ among the three groups (~30% in each group). To assess endothelial function, dilation of isolated MCAs in response to of acetylcholine (10^-8^ to 10^-5^ mol/L) and ATP (10^-7^ mol/L) were obtained in the absence and presence of L-NAME (3x10^-4^ mol/L, for 30 min). To assess endothelium-independent vasodilation, responses to the NO donor sodium nitroprusside (SNP, 10^-7^ mol/L) were assessed. At the end of each experiment the vessels were superfused with Ca^2+^-free Krebs’ buffer containing nifedipine (10^-5^ mol/L) to achieve maximal vasodilatation.

*Quantitative real-time RT-PCR*

A quantitative real time RT-PCR technique was used to analyze mRNA expression for the nitric oxide synthases eNOS and nNOS (*Nos3* and *Nos1*, respectively*)*, arginases (*Arg1, Arg2*; which regulate NO synthase activity and were proposed to contribute to endothelial dysfunction in aging([Ungvari *et al.* 2010](#_ENREF_20))), NADPH oxidases (*Nox1, Nox2*) and superoxide dismutases (*Sod1, Sod2*) in cortical samples using validated TaqMan probes (Applied Biosystems) and a Strategen MX3000 platform, as previously reported([Toth *et al.* 2015a](#_ENREF_15)). In brief, total RNA was isolated with a Mini RNA Isolation Kit (Zymo Research, Orange, CA) and was reverse transcribed using Superscript III RT (Invitrogen) as described previously([Toth *et al.* 2015a](#_ENREF_15)). Quantification was performed using the efficiency-corrected ΔΔCq method. The relative quantities of the reference genes *Hprt, Ywhaz, B2m*, *Actb* and S18 were determined and a normalization factor was calculated based on the geometric mean for internal normalization. Fidelity of the PCR reaction was determined by melting temperature analysis and visualization of the product on a 2% agarose gel.

*Assessment of the effect of in vitro treatment with SS-31 on age-related increases in mtROS production in cultured cerebromicrovascular endothelial cells*

To confirm the direct anti-oxidative endothelium-protective effect of SS-31 *in* *vitro*, we assessed the effect of SS-31 on mtROS production in cultured primary cerebromicrovascular endothelial cells (CMVECs). The establishment and characterization of the cell strains used has been recently reported([Tucsek *et al.* 2012](#_ENREF_19); [Ungvari *et al.* 2012](#_ENREF_24); [Csiszar *et al.* 2014](#_ENREF_3); [Banki *et al.* 2015](#_ENREF_2)).

In brief, to establish primary cultures of CMVECs, the brains of male 3 and 24 month old F344xBN rats (obtained from the National Institute on Aging) were removed aseptically, rinsed in ice cold PBS and minced into ≈1 mm squares. The tissue was washed twice in ice cold 1X PBS by low-speed centrifugation (50g, 2-3 min). The diced tissue was digested in a solution of collagenase (800U/g tissue), hyaluronidase (2.5U/g tissue) and elastase (3U/g tissue) in 1ml PBS/100mg tissue for 45 min at 37 °C in a rotating humid incubator. The digested tissue was passed through a 100um cell strainer. The single cell lysate was centrifuged for 2min at 70g. After removing the supernatant the pellet was washed twice in cold PBS supplemented with 2.5 % fetal calf serum (FCS) and the suspension centrifuged at 300g for 5 min at 4C. To create an endothelial cell enriched fraction the cell suspension was centrifuged using an OptiPrep gradient solution (Axi-Shield, PoC, Norway). Briefly, the cell pellet was resuspended in Hanks' balanced salt solution (HBSS) and mixed with 40% iodixanol thoroughly (final concentration: 17% (w/v) iodixanol solution; ρ = 1.096 g/ml). 2 ml of HBSS was layered on top and centrifuged at 400 *g* for 15 min at 20°C. Endothelial cells, which banded at the interface between HBSS and the 17% iodixanol layer, were collected. The endothelial cell enriched fraction was incubated for 30 min at 4 ^o^C in the dark with anti-CD31/PE (BD BD Biosciences, San Jose, CA, USA), anti-MCAM/FITC (BD Biosciences, San Jose, CA, USA). After washing the cells twice with MACS Buffer (Milltenyi Biotech, Cambridge, MA, USA) anti-FITC magnetic beads labeled with anti-PE magnetic bead labeled secondary antibodies were used for 15 min at room temperature. Endothelial cells were collected by magnetic separation using the MACS LD magnetic separation columns according to the manufacturer’s guidelines (Milltenyi Biotech, Cambridge, MA, USA). The endothelial fraction was cultured on fibronectin coated plates in Endothelial Growth Medium (Cell Application, San Diego, CA, USA) for 10 days. Endothelial cells were phenotypically characterized by flow cytometry (GUAVA 8HT, Merck Millipore, Billerica, MA, USA). Briefly, antibodies against five different endothelial specific markers were used (anti-CD31-PE, anti-erythropoietin receptor-APC, anti-VEGF R2-PerCP, anti-ICAM-fluorescein, anti-CD146-PE) and isotype specific antibody labeled fractions served as negative controls. Flow cytometric analysis showed that after the third cycle of immunomagnetic selection there were virtually no CD31-, CD146-, EpoR- and VEGFR2- cells in the resultant cell populations. All antibodies were purchased from R&D Systems (R&D Systems, Minneapolis, MN, USA).

To assess the direct effects of SS-31 on endothelial ROS production, primary CMVECs derived from young and aged rats were treated with SS-31 *in vitro* (10^-9^ to 10^-5^ mol/L, for 24 h or 10^-5^ mol/L, for 1 to 24 h). Following the treatment period mitochondrial O_2_^.-^ production in CMVECs was measured using MitoSOX Red (Invitrogen, Carlsbad CA), a mitochondrion-specific hydroethidine-derivative fluorescent dye([Ungvari *et al.* 2007](#_ENREF_22); [Ungvari *et al.* 2009](#_ENREF_21); [Csiszar *et al.* 2012a](#_ENREF_5); [Csiszar *et al.* 2012b](#_ENREF_6); [Ungvari *et al.* 2013](#_ENREF_23)), as previously reported([Csiszar *et al.* 2008](#_ENREF_4)). In brief, CMVECs were loaded with MitoSox (3 µmol/L for 30 min) followed by washout and an equilibration period of 20 min. MitoSOX fluorescence was assessed by ﬂow cytometry as previously reported([Csiszar *et al.* 2012a](#_ENREF_5); [Csiszar *et al.* 2012b](#_ENREF_6)) using the Guava easyCyte 8HT flow-cytometer (Millipore, Hayward, CA).

*Seahorse*

To substantiate the endothelium-protective effect of SS-31, we performed real-time measurements of the oxygen consumption rate (OCR; a marker of oxidative phosphorylation) in young and aged CMVECs after treatment with SS-31 (10^-5^ mol/L, for 48 h) using a Seahorse XF96 extracellular flux analyzer.

*Statistical analysis*

Data were analyzed by one-way analysis of variance (ANOVA) followed by Tukey’s post-hoc test. Principal component analysis, followed by MANOVA and the PCA-biplot approach were used to analyze the gait data. A p value less than 0.05 was considered statistically significant. Data are expressed as mean±S.E.M.

**References**

Ammassari-Teule M , Caprioli A (1985). Spatial learning and memory, maze running strategies and cholinergic mechanisms in two inbred strains of mice. *Behav Brain Res*. **17**, 9-16.

Banki E, Sosnowska D, Tucsek Z, Gautam T, Toth P, Tarantini S, Tamas A, Helyes Z, Reglodi D, Sonntag WE, Csiszar A , Ungvari Z (2015). Age-related decline of autocrine pituitary adenylate cyclase-activating polypeptide impairs angiogenic capacity of rat cerebromicrovascular endothelial cells. *J Gerontol A Biol Sci Med Sci*. **70**, 665-674.

Csiszar A, Gautam T, Sosnowska D, Tarantini S, Banki E, Tucsek Z, Toth P, Losonczy G, Koller A, Reglodi D, Giles CB, Wren JD, Sonntag WE , Ungvari Z (2014). Caloric restriction confers persistent anti-oxidative, pro-angiogenic, and anti-inflammatory effects and promotes anti-aging miRNA expression profile in cerebromicrovascular endothelial cells of aged rats. *Am J Physiol Heart Circ Physiol*. **307**, H292-306.

Csiszar A, Labinskyy N, Perez V, Recchia FA, Podlutsky A, Mukhopadhyay P, Losonczy G, Pacher P, Austad SN, Bartke A , Ungvari Z (2008). Endothelial function and vascular oxidative stress in long-lived GH/IGF-deficient Ames dwarf mice. *Am J Physiol Heart Circ Physiol*. **295**, H1882-1894.

Csiszar A, Podlutsky A, Podlutskaya N, Sonntag WE, Merlin SZ, Philipp EER, Doyle K, Davila A, Recchia FA, Ballabh P, Pinto JT , Ungvari Z (2012a). Testing the oxidative stress hypothesis of aging in primate fibroblasts: is there a correlation between species longevity and cellular ROS production? . *J Gerontol A Biol Sci Med Sci*. **67**, 841-852.

Csiszar A, Sosnowska D, Wang M, Lakatta EG, Sonntag WE , Ungvari Z (2012b). Age-associated proinflammatory secretory phenotype in vascular smooth muscle cells from the non-human primate Macaca mulatta: reversal by resveratrol treatment. *J Gerontol A Biol Sci Med Sci*. **67**, 811-820.

Dai DF, Chen T, Szeto H, Nieves-Cintron M, Kutyavin V, Santana LF , Rabinovitch PS (2011). Mitochondrial targeted antioxidant peptide ameliorates hypertensive cardiomyopathy. *J Am Coll Cardiol*. **58**, 73-82.

Hall JL , Berman RF (1995). Juvenile experience alters strategies used to solve the radial arm maze in rats. *Psychobiology*. **23**, 195. doi:110.3758/BF03332022.

Kazama K, Anrather J, Zhou P, Girouard H, Frys K, Milner TA , Iadecola C (2004). Angiotensin II impairs neurovascular coupling in neocortex through NADPH oxidase-derived radicals. *Circ Res*. **95**, 1019-1026.

MacLaren DA, Santini JA, Russell AL, Markovic T , Clark SD (2014). Deficits in motor performance after pedunculopontine lesions in rats - impairment depends on demands of task. *The European journal of neuroscience*.

Maho C, Dutrieux G , Ammassari-Teule M (1988). Parallel modifications of spatial memory performances, exploration patterns, and hippocampal theta rhythms in fornix-damaged rats: reversal by oxotremorine. *Behav Neurosci*. **102**, 601-604.

Shukitt-Hale B, McEwen JJ, Szprengiel A , Joseph JA (2004). Effect of age on the radial arm water maze-a test of spatial learning and memory. *Neurobiol Aging*. **25**, 223-229.

Springo Z, Tarantini S, Toth P, Tucsek Z, Koller A, Sonntag WE, Csiszar A , Ungvari Z (2015). Aging Exacerbates Pressure-Induced Mitochondrial Oxidative Stress in Mouse Cerebral Arteries. *J Gerontol A Biol Sci Med Sci*. **70**, 1355-1359.

Tarantini S, Hertelendy P, Tucsek Z, Valcarcel-Ares MN, Smith N, Menyhart A, Farkas E, Hodges E, Towner R, Deak F, Sonntag WE, Csiszar A, Ungvari Z , Toth P (2015). Pharmacologically-induced neurovascular uncoupling is associated with cognitive impairment in mice. *J Cereb Blood Flow Metab*. **35**, 1871-1881.

Toth P, Tarantini S, Ashpole NM, Tucsek Z, Milne GL, Valcarcel-Ares NM, Menyhart A, Farkas E, Sonntag WE, Csiszar A , Ungvari Z (2015a). IGF-1 deficiency impairs neurovascular coupling in mice: implications for cerebromicrovascular aging. *Aging Cell*. **14**, 1034-1044.

Toth P, Tarantini S, Springo Z, Tucsek Z, Gautam T, Giles CB, Wren JD, Koller A, Sonntag WE, Csiszar A , Ungvari Z (2015b). Aging exacerbates hypertension-induced cerebral microhemorrhages in mice: role of resveratrol treatment in vasoprotection. *Aging Cell*. **14**, 400-408.

Toth P, Tarantini S, Tucsek Z, Ashpole NM, Sosnowska D, Gautam T, Ballabh P, Koller A, Sonntag WE, Csiszar A , Ungvari ZI (2014). Resveratrol treatment rescues neurovascular coupling in aged mice:role of improved cerebromicrovascular endothelial function and down-regulation of NADPH oxidas. *Am J Physiol Heart Circ Physiol*. **306**, H299-308.

Toth P, Tucsek Z, Sosnowska D, Gautam T, Mitschelen M, Tarantini S, Deak F, Koller A, Sonntag WE, Csiszar A , Ungvari Z (2013). Age-related autoregulatory dysfunction and cerebromicrovascular injury in mice with angiotensin II-induced hypertension. *J Cereb Blood Flow Metab*. **33**, 1732-1742.

Tucsek Z, Gautam T, Sonntag WE, Toth P, Saito H, Salomao R, Szabo C, Csiszar A , Ungvari Z (2012). Aging Exacerbates Microvascular Endothelial Damage Induced by Circulating Factors Present in the Serum of Septic Patients. *J Gerontol A Biol Sci Med Sci*.

Ungvari Z, Kaley G, de Cabo R, Sonntag WE , Csiszar A (2010). Mechanisms of vascular aging: new perspectives. *J Gerontol A Biol Sci Med Sci*. **65**, 1028-1041.

Ungvari Z, Labinskyy N, Mukhopadhyay P, Pinto JT, Bagi Z, Ballabh P, Zhang C, Pacher P , Csiszar A (2009). Resveratrol attenuates mitochondrial oxidative stress in coronary arterial endothelial cells. *Am J Physiol Heart Circ Physiol*. **297**, H1876-1881.

Ungvari Z, Orosz Z, Labinskyy N, Rivera A, Xiangmin Z, Smith K , Csiszar A (2007). Increased mitochondrial H2O2 production promotes endothelial NF-kappaB activation in aged rat arteries. *Am J Physiol Heart Circ Physiol*. **293**, H37-47.

Ungvari Z, Podlutsky A, Sosnowska D, Tucsek Z, Toth P, Deak F, Gautam T, Csiszar A , Sonntag WE (2013). Ionizing Radiation Promotes the Acquisition of a Senescence-Associated Secretory Phenotype and Impairs Angiogenic Capacity in Cerebromicrovascular Endothelial Cells: Role of Increased DNA Damage and Decreased DNA Repair Capacity in Microvascular Radiosensitivity. *J Gerontol A Biol Sci Med Sci*.

Ungvari Z, Tucsek Z, Sosnowska D, Toth P, Gautam T, Podlutsky A, Csiszar A, Losonczy G, Valcarcel-Ares MN , Sonntag WE (2012). Aging-Induced Dysregulation of Dicer1-Dependent MicroRNA Expression Impairs Angiogenic Capacity of Rat Cerebromicrovascular Endothelial Cells. *J Gerontol A Biol Sci Med Sci*.
